# Optimization of salicylic acid and chitosan treatment for bitter secoiridoid and xanthone glycosides production in shoot cultures of *Swertia paniculata* using response surface methodology and artificial neural network

**DOI:** 10.1186/s12870-020-02410-7

**Published:** 2020-05-19

**Authors:** Prabhjot Kaur, R. C. Gupta, Abhijit Dey, Tabarak Malik, Devendra Kumar Pandey

**Affiliations:** 1 Department of Biotechnology, Lovely Faculty of Technology and Sciences, Lovely Professional University, Phagwara, Punjab 144411 India; 2 Department of Botany, Punjabi University, Patiala, Punjab 147002 India; 3 Department of Life Sciences, Presidency University, Kolkata, India; 4 Department of Biochemistry, College of Medicine and Health Sciences, University of Gondar, Gondar, Ethiopia

**Keywords:** *Swertia*, Elicitors, Secoiridoids, Mangiferin, Response surface methodology, Artificial neural network

## Abstract

**Background:**

In this study, response surface methodology (RSM) and artificial neural network (ANN) was used to construct the predicted models of linear, quadratic and interactive effects of two independent variables viz. salicylic acid (SA) and chitosan (CS) for the production of amarogentin (I), swertiamarin (II) and mangiferin (III) from shoot cultures of *Swertia paniculata* Wall*.* These compounds are the major therapeutic metabolites in the *Swertia* plant, which have significant role and demand in the pharmaceutical industries.

**Results:**

Present study highlighted that different concentrations of SA and CS elicitors substantially influenced the % yield of (I), (II) and (III) compounds in the shoot culture established on modified ½ MS medium (supplemented with 2.22 mM each of BA and KN and 2.54 mM NAA). In RSM, different response variables with linear, quadratic and 2 way interaction model were computed with five-factor-three level full factorial CCD. In ANN modelling, 13 runs of CCD matrix was divided into 3 subsets, with approximate 8:1:1 ratios to train, validate and test. The optimal enhancement of (I) (0.435%), (II) (4.987%) and (III) (4.357%) production was achieved in 14 days treatment in shoot cultures of *S. paniculata* elicited by 9 mM and 12 mg L^− 1^ concentrations (SA) and (CS).

**Conclusions:**

In optimization study, (I) show 0.170–0.435%; (II) display 1.020–4.987% and (III) upto 2.550–4.357% disparity with varied range of SA (1–20 mM) and CS (1–20 mg L^− 1^). Overall, optimization of elicitors to promote secoiridoid and xanthone glycoside production with ANN modeling (*r*^*2*^ = 100%) offered more significant results as compared to RSM (*r*^*2*^ = 99.8%).

**Graphical abstract:**

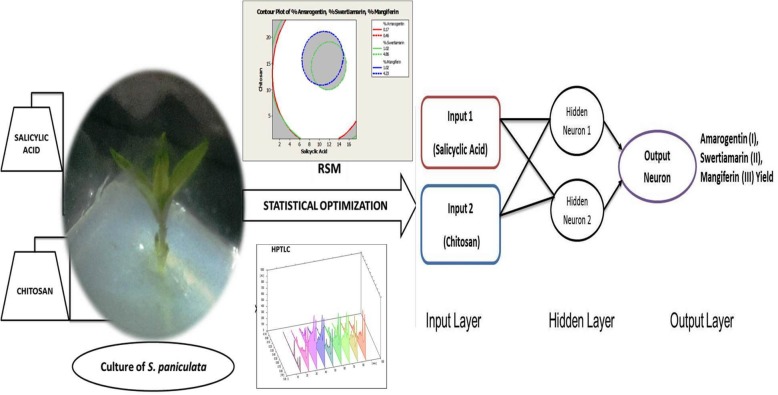

## Background

*Swertia* (Family: Gentianaceae) is a diverse genus with worldwide distribution at the north temperate areas of Asia, America, Europe and Africa [1]. This genus has been cited as an effective herbal medicine in Indian traditional (Ayurvedic, Unani and Sidha) and British Pharmacopoeias. Presence of bio-active compounds, viz. amarogentin (the bitterest compound with the bitter index of 58,000,000), swertiamarin and mangiferin were found to be responsible for wide-range of therapeutic potential [2]. *S. chirata* (Roxb. ex Fleming) H. Karst. is used as a principal component in several commercial herbal/polyherbal formulations. *S. paniculata* Wall. is generally used as a substitute of *S. chirata* in many herbal formulations [3]. It has been reported long back that *S. paniculata* contained medicinally important bioactive compounds [3–6], which have been supported by the recent studies [7, 8]. Extracts from *S. paniculata* are used as a bitter tonic in the Indian System of Medicine and in the treatment of some mental disorders [4]. Negi et al. [5] reported the anti-diabetic activity of *S. paniculata* and found 51.0% decreased level of blood sugar in alloxan induced rats. Biological activities of *S. paniculata* are due to the presence of xanthone glycosides and bitter secoiridoids [3–8]. Ever-increasing industrial demand, overharvesting, low seed germination rate (2–3%), and other anthropogenic intrusions adversely disturbed the wild populations of various *Swertia* species. Several *Swertia* species are listed as critically endangered and thus the gap between the commercial demand and supply of *Swertia* herb is continuously growing. Once a particular plant is commercialized, subsequently that species become endangered soon due to extensive overharvesting. Government agencies like the World Health Organization (WHO) and European Medicines Agency (EMA) developed some principles already to moderate the threatened status of medicinal plants. Advancement in plant biotechnology holds great assurance for the conservation and production of improved variety of medicinally important rare and endangered flora and large-scale production of plant derived secondary metabolites. In a variety of medicinal plants, tissue culture technique has been successfully proven as a potential alternative strategy for the production of valuable bio-active compounds with clinical significance [9]. In *Swertia* spp., very few reports are available on the in vitro strategies that are used to enhance growth and secondary metabolite production utilizing hairy/adventitious root cultures, rhizobacteria etc. [10–12]. Hitherto, there is no report on the effects of salicylic acid (SA) and chitosan (CS) on the production of secoiridoid and xanthone glycosides in *S. paniculata* shoot cultures.

Elicitors can induce biotic or abiotic stresses in plants that further enhance specific enzymatic reactions and induces subset of genes linked to the signaling pathways of desired secondary metabolites [13]. Among abiotic and biotic elicitors, salicylic acid (SA) and o-hydroxybenzoic acid is an important endogenous signal molecule that is well known to develop systemic acquired resistance (SAR) and induced systemic resistance (ISR) in plants [14]. SA signaling pathways facilitates the SAR that protects plants against pathogens and consequently produces a pathogen related proteins (PRPs). SA is also known to induce reactive oxygen species (ROS) production as a part of the defense system in plants. Numerous studies displayed the exogenous effects of SA on the elicitation of different classes of phenolic compounds [15–18]. Remarkably, chitosan (CS) is non-toxic, cost-effective and the second most abundant polycationic biopolymer that is well documented in the elicitation of in vitro production of high-valued secondary metabolites such as aromatic amino acids, phenylpropanoids, plumbagin, tannins, triterpenoids, xanthones, secoiridoids etc. [19–22].

It is well-known that only the optimal levels of elicitor and precursor concentration increases the in vitro production of desired secondary metabolites. In optimization of medium for metabolite production, Central Composite Design (CCD) and Box-Behnken design (BBD) are the most commonly used designs that obtain optimal response from statistical mathematical modeling of Response Surface Methodology (RSM) [23, 24]. Statistically, RSM uses symmetrical experimental designs, explores the effects of single or multiple response variables and optimize these variables to get the best possible response [25]. On the other hand, Artificial Neural Network (ANN) is an information processing model based on the nonlinear weighted sum statistical data modeling tools. ANN is inspired by the way of biological neuron network that discovers a complex connection between the responses and predicted variables. In contrast to RSM, ANN is a more accurate method of interpolation, prediction, and validation [26].

Amarogentin, swertiamarin, and mangiferin are considered as the three prime phytochemical markers of the genus *Swertia*. Therefore, it is of great interest to optimize the in vitro production of these compounds. Optimized micropropagation techniques have the potential to be beneficial to encounter the demand for medicinal plants used in pharmaceutical industries. Plant growth regulators have been reported for high-frequency regeneration and in vitro production of bioactive compounds [27].

Present work has been devoted to the optimization of effects of SA and CS on in vitro production of amarogentin, swertiamarin, and mangiferin in *S. paniculata*. The optimization process was accomplished with full-factorial CCD using multivariate response surface analysis and was then compared with ANN model.

## Results

### Organogenesis

In this study, the combinations of BAP (6-Benzylaminopurine), KN (Kinetin) and NAA (1-Naphthaleneacetic acid) were used for organogenesis. High rate of shoot regeneration and elongation was achieved with ½ MS media supplemented with 2.22 μM BAP, KN and 2.60 μM NAA after 6 weeks. Average length of shoot with different concentrations of shoot inducing medium (SIM) are presented in Table 1.

**Table 1:** Average length of shoot with different concentrations of shoot inducing medium (SIM)

Types of media	Plant growth regulators (μM)	Mean shoot length (cm) (After 4 weeks)	Mean shoot length (cm) (After 6 weeks)
½ MS	Control	0.00	0.00
	BAP + KN		
	(2.22 + 2.22)	1.2 ± 0.1^a^	1.4 ± 0.2^a^
	(4.44 + 4.44)	0.6 ± 0.2^a^	0.7 ± 0.1^a^
	BAP + KN + NAA		
	(2.22 + 2.22 + 2.60)	3.2 ± 0.2^a^	**5.8 ± 0.4**^**a**^
	(4.44 + 4.44 + 5.20)	1.6 ± 0.1^a^	2.7 ± 0.2^a^
MS	Control	0.00	0.00
	BAP + KN		
	(2.22 + 2.22)	0.00	0.00
	(4.44 + 4.44)	0.00	0.00
	BAP + KN + NAA		
	(2.22 + 2.22 + 2.60)	0.00	1.3 ± 0.1^a^
	(4.44 + 4.44 + 5.20)	0.00	0.00

All the values are represented in means± standard deviation (where *n* = 3)

#### Experimental design and statistical optimization by RSM

In present study, treatment of shoot cultures with optimal concentration of SA (below 10 mM) and CS (above 10 mg/L) increases the yield of amarogentin, swertiamarin and mangiferin as compared to the control sets of cultures (shoots which were not treated with elicitors). Maximum % yield of amarogentin (0.435%), swertiamarin (4.987%) and mangiferin (4.357%) was observed with cultures treated with 9 mM SA and 12 mg/L CS after 14 days that were 0.25, 3.0 and 2.2 times more as compared to controlled cultures, respectively.

**Fig. 1:**
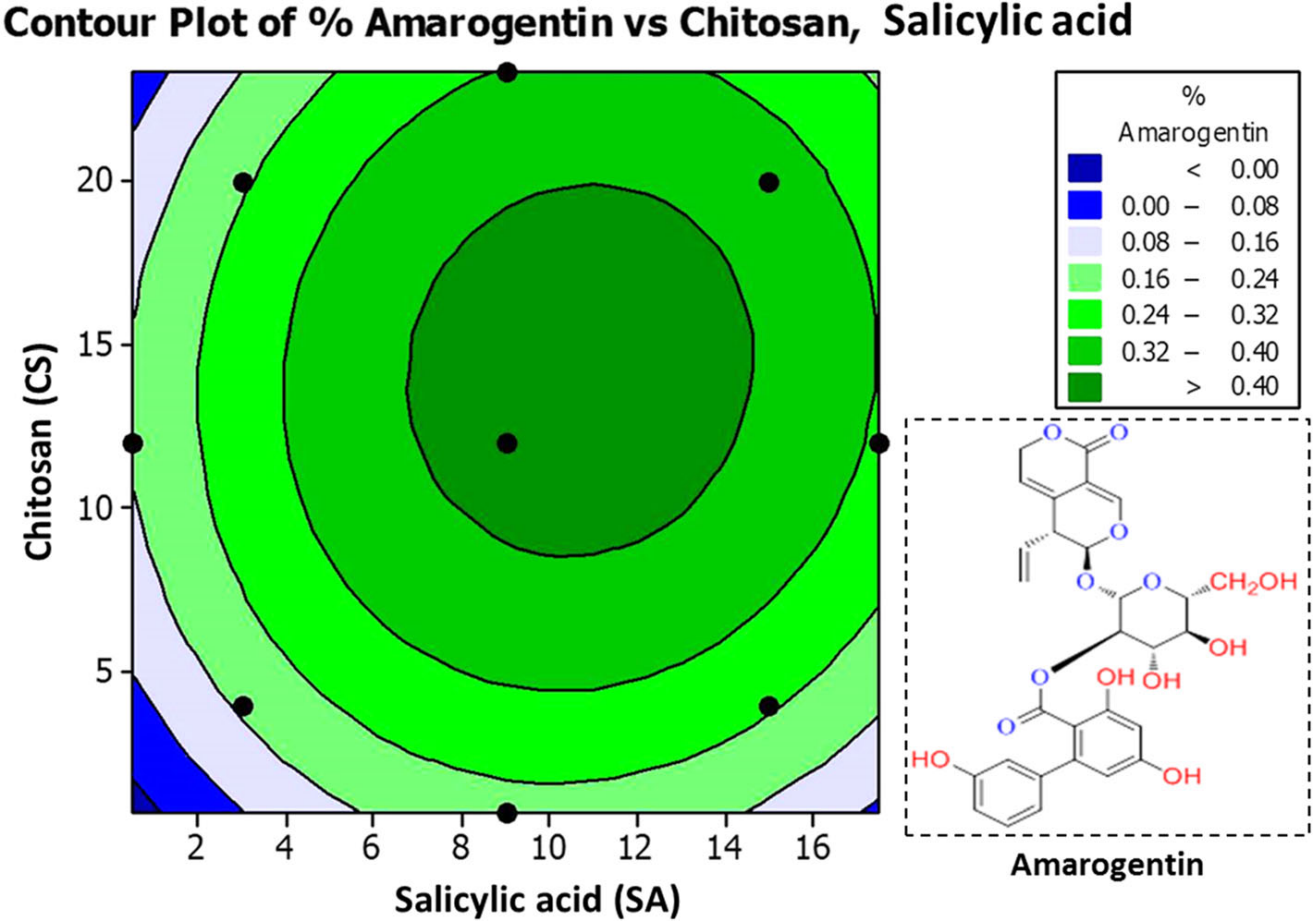
Contour plot for amarogentin (%) at different concentrations of salicylic acid and chitosan elicitors

**Fig. 2:**
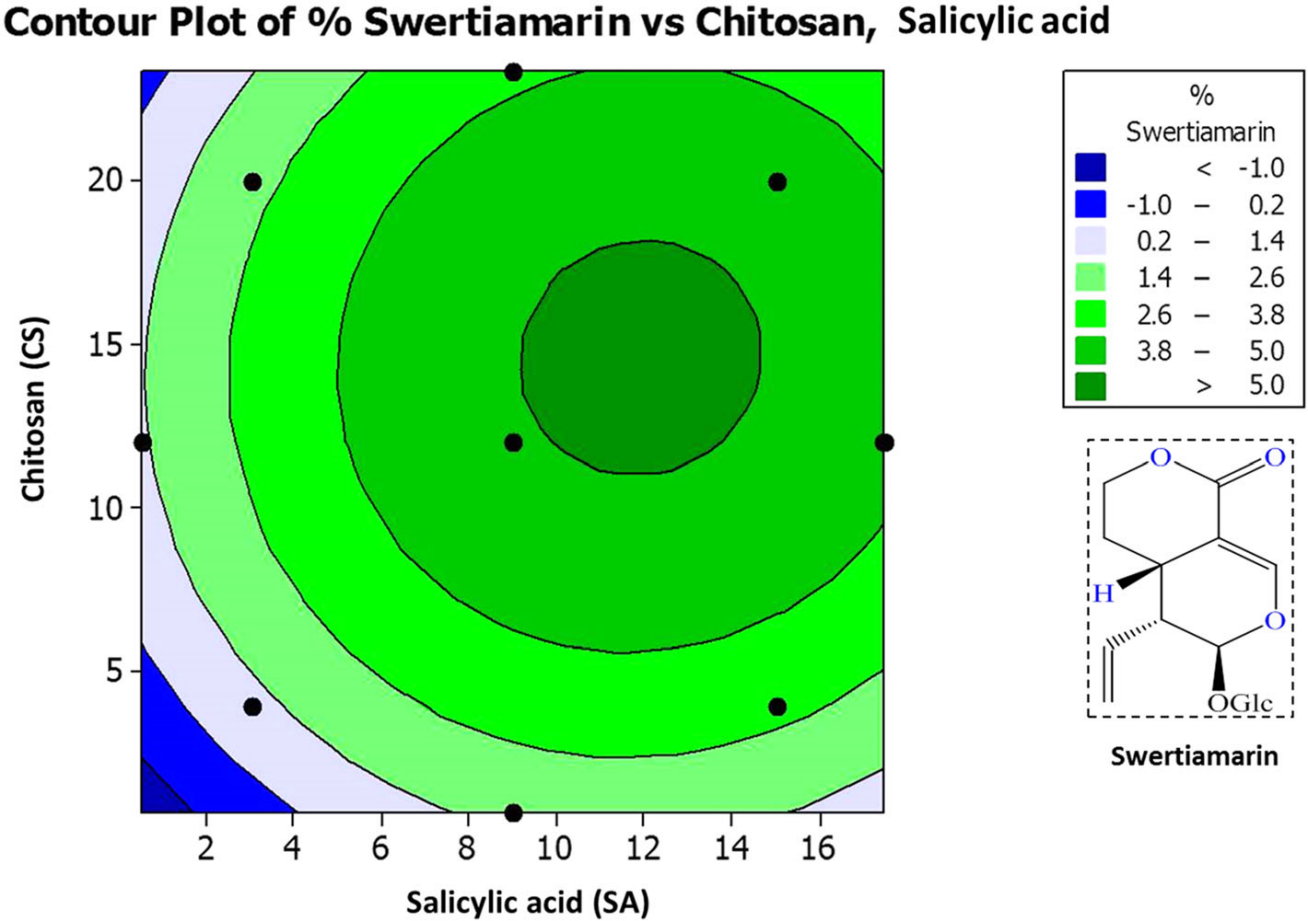
Contour plot for swertiamarin (%) at different concentrations of salicylic acid and chitosan elicitors

**Fig. 3:**
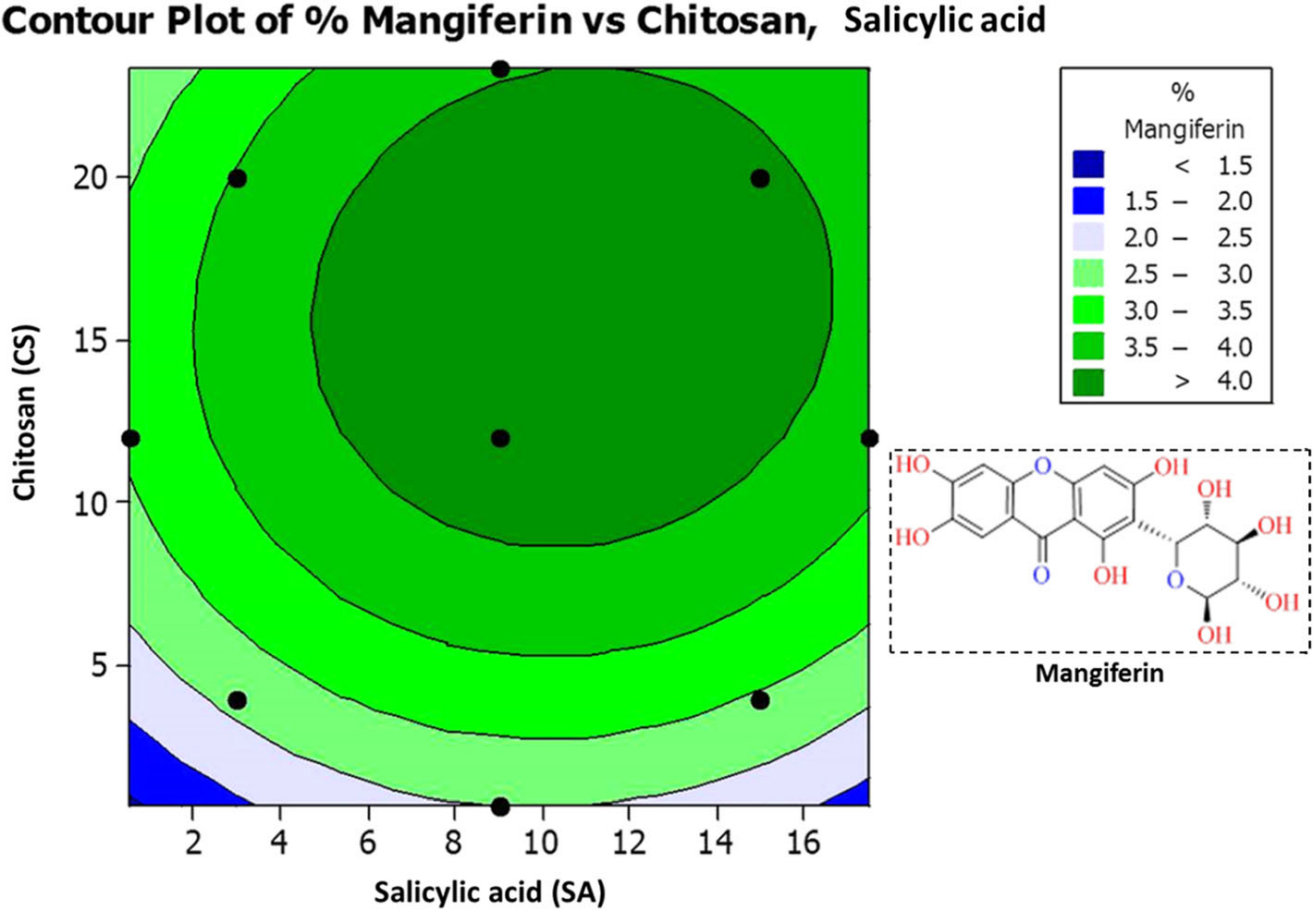
Contour plot for mangiferin (%) at different concentrations of salicylic acid and chitosan elicitors

For statistical optimization by RSM, Central composite design (CCD) was used to optimize the two variables (*α* = 1.41) and all factors were studied at five different levels (−*a*, − 1, 0, + 1, +*a*). With CCD, experimental values of amarogentin (I), swertiamarin (II), and mangiferin (III) in respect to the effects of SA and CS are summarized in Table 2. Response surface regression and Analysis of variance (ANOVA) for % yield of (I), (II) and (III) compounds were calculated, and are summarized in Additional file 1: Table S1 and S2. Higher *Fisher* distribution (*F t*est) for (I) (1213.5), (II) (1818.51), and (III) (668.11) indicates the adequacy of the model. In this experimentation, *p<* 0.001 was considered as very significant, but *p<* 0.05 as significant values. In this model, Lack-of-Fit was not significant (*p>* 0.05) as it represents lesser F-values for (I) (1.51), (II) (0.55), and (III) (0.82). Significant regression and non-significant Lack-of-Fit confirms the adequacy and well-fitness of mathematical modeling with experiment data [25]. *R*^*2*^ values obtained from (I) (99.64%), (II) (99.82%) and (III) (99.51%) also revealed the good correlation between response values and independent variables. Predicted values obtained through this design were used to draw the contour plots of SA and CS versus yield % of amarogentin (Fig. 1), swertiamarin (Fig. 2), and mangiferin (Fig. 3) compounds.

**Table 2:** Experimental and predicted values for secoiridoid and xanthone glycoside yield (%) optimized with central composite design (CCD)

Run order	Treatment		Secoiridoid Yield (%)						Xanthone Yield (%)		
	SA (mM) (A)	CS (mg L^− 1^) (B)	Amarogentin (I)			Swertiamarin (II)			Mangiferin (III)		
			Experimental	Predicted		Experimental	Predicted		Experimental	Predicted	
				RSM	ANN		RSM	ANN		RSM	ANN
1	3.000	4.000	0.170	0.173	0.170	1.020	1.064	1.020	2.610	2.640	2.610
2	15.000	4.000	0.247	0.249	0.250	2.860	2.905	2.860	2.910	2.951	2.910
3	3.000	20.000	0.225	0.230	0.225	2.235	2.241	2.235	3.550	3.533	3.550
4	15.000	20.000	0.350	0.354	0.350	4.450	4.457	4.450	4.110	4.104	4.110
5	0.514	12.000	0.172	0.167	0.171	1.320	1.294	1.320	3.080	3.075	3.080
6	17.485	12.000	0.312	0.308	0.311	4.190	4.163	4.193	3.720	3.699	3.720
7	9.000	0.686	0.210	0.208	0.210	1.695	1.641	1.695	2.550	2.504	2.550
8	9.000	23.313	0.330	0.323	0.330	3.570	3.570	3.572	3.930	3.950	3.932
9	9.000	12.000	0.430	0.428	0.427	4.860	4.870	4.830	4.254	4.288	4.253
10	9.000	12.000	0.435	0.428	0.427	4.830	4.870	4.830	4.237	4.288	4.253
11	9.000	12.000	0.425	0.428	0.427	4.875	4.870	4.867	4.320	4.288	4.283
12	9.000	12.000	0.430	0.428	0.427	4.987	4.870	4.970	4.357	4.288	4.329
13	9.000	12.000	0.420	0.428	0.427	4.800	4.870	4.867	4.275	4.288	4.253

Different response variables with linear, quadratic and 2-way interaction models were computed according to the quadratic eq. (1), mentioned above and all the variables were significant (Additional file 1: Table S1 and S2). Multiple regression equation based on the second order kinetics was calculated for the predicted response values (%) of amarogentin (Y_1_), swertiamarin (Y_2_), and mangiferin (Y_3_).

1 $$ {\mathrm{Y}}_1=0.42800+0.05000\ \left(\mathrm{A}\right)+0.04096\ \left(\mathrm{B}\right)\hbox{-} 0.09500\ \left({\mathrm{A}}^2\right)\hbox{-} 0.08100\ \left({\mathrm{B}}^2\right)+0.01200\ \left(\mathrm{A}\mathrm{B}\right) $$

2 $$ {\mathrm{Y}}_2=4.87040+1.01422\ \left(\mathrm{A}\right)+0.68208\ \left(\mathrm{B}\right)\hbox{-} 1.07083\ \left({\mathrm{A}}^2\right)\hbox{-} 1.13207\ \left({\mathrm{B}}^2\right)+00.09375\ \left(\mathrm{A}\mathrm{B}\right) $$

3 $$ {\mathrm{Y}}_3=4.28860+0.22064\ \left(\mathrm{A}\right)+0.51145\ \left(\mathrm{B}\right)\hbox{-} 0.45055\ \left({\mathrm{A}}^2\right)\hbox{-} 0.53055\ \left({\mathrm{B}}^2\right)+0.06500\ \left(\mathrm{A}\mathrm{B}\right) $$

Where A and B represent salicylic acid and chitosan that were used as significant response variables to get the maximum yield of (I), (II), and (III) in cultured plants of *S. paniculata.* In the present experiment, amarogentin, swertiamarin, and mangiferin showed 0.170–0.435%, 1.020–4.987%, and 2.550–4.357% disparity respectively. Linear regression coefficient of both the independent variables (A and B) showed positive effect on the yield of (I), (II), and (III) (Additional file 1: Table S1). Linear coefficient values revealed that SA effects swertiamarin yield (1.01422) maximum followed by mangiferin yield (0.22064) but showed lesser impact on amarogentin content (0.05000). On the other hand, CS also impacted swertiamarin yield (0.68208) maximum followed by mangiferin yield (0.51145) with lesser impact on amarogentin content (0.04096). Out of the two independent variables (A and B), SA showed more impact on the swertiamarin and amarogentin yield but CS demonstrated comparatively high impact on mangiferin yield. As shown in Additional file 1: Table S1 and S2, linear and quadratic effects of A and B variables were found to be very significant (*p<* 0.001) for (I), (II), and (III) while interaction of both the variables (A and B) showed only significant effects (*p*< 0.05).

**Fig. 4:**
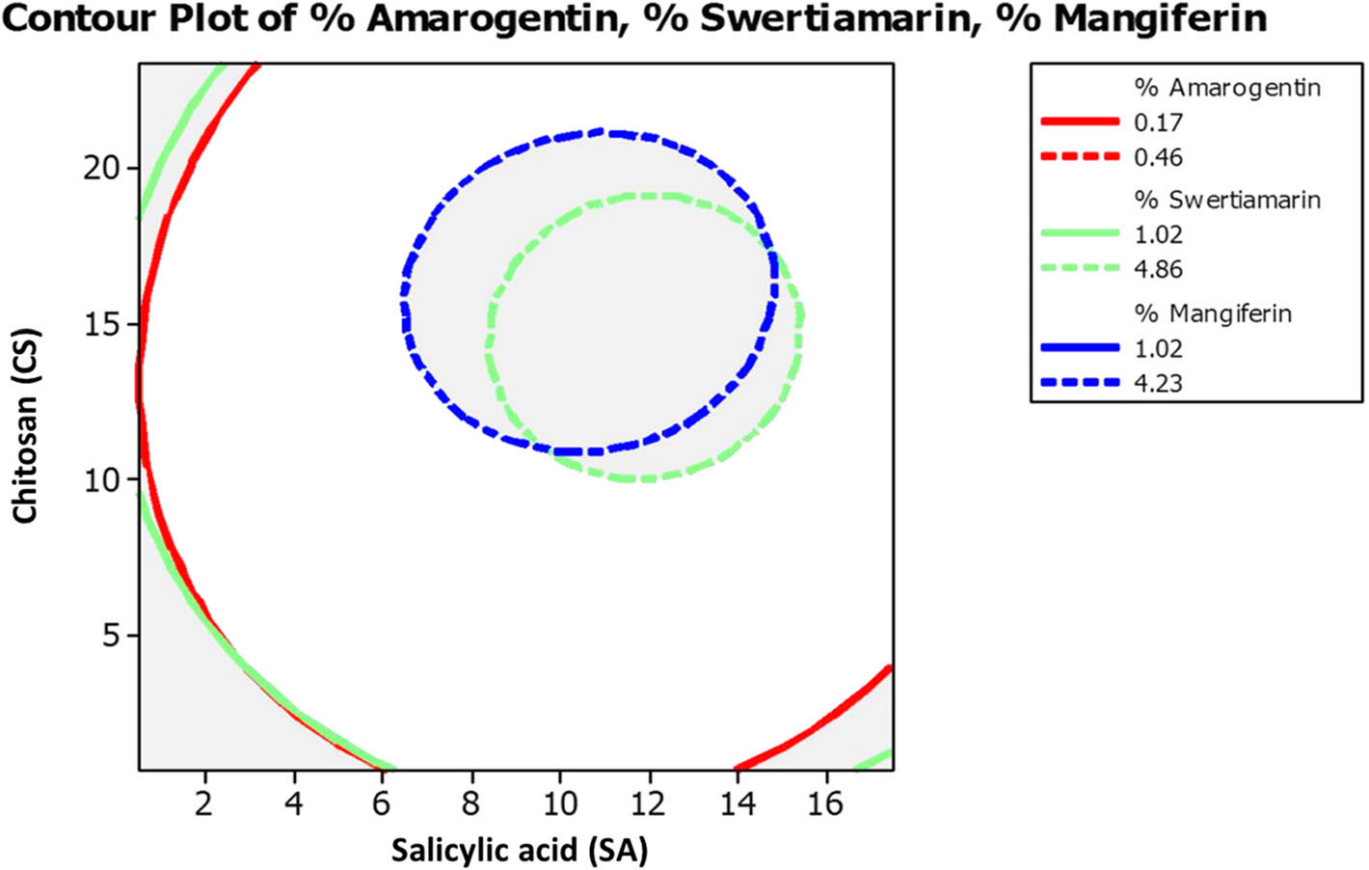
Comparative contour plot for amarogentin, swertiamarin and mangiferin (%) at different concentrations of salicylic acid and chitosan elicitors

As depicted in contour plots (Figs. 1, 2, 3, and 4), optimal values obtained for SA and CS are 9 mM and 12 mg L^− 1^ respectively that yielded maximum amount of amarogentin (0.435%), swertiamarin (4.987%), and mangiferin (4.357%). Experimental values were very close to the predicted values (Table 2), therefore this mathematical model was appropriately developed for the statistical optimization of the effects of SA and CS on the yield of secoiridoid (amarogentin and swertiamarin) and xanthone (mangiferin) glycosides.

#### ANN modeling

**Fig. 5:**
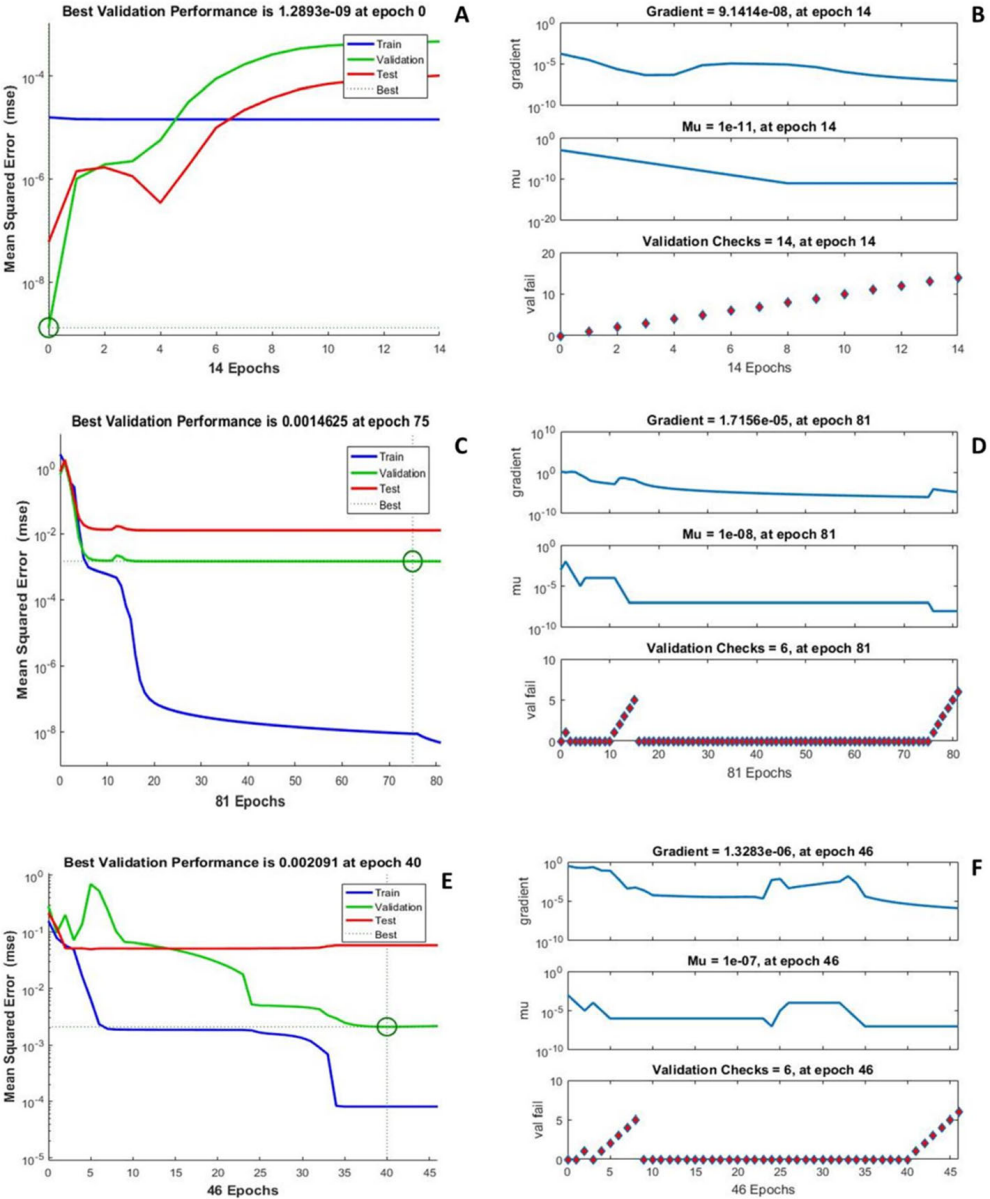
Performance data obtained (ANN) over entire training data and gradient loss for amarogentin (**a, b**); swertiamarin (**c, d**); mangiferin (**e, f**)

In the present study, ANN adopted back propagation algorithm during training phase and was developed into 3 layers: input layer (A and B), hidden layer and output layer (% yield of amarogentin, swertiamarin, and mangiferin). In ANN modeling, 13 runs of CCD matrix were divided into 3 subsets, with approximate 8:1:1 ratio to train, validate and test. Figure 5 depicted the performance data obtained over entire training data and fitted at best epochs for validation data being represented. Similarly, gradient loss and training state achieved over entire ANN training is explained with the help of Fig. 5. Best validation performance for the optimization of amarogentin (I), swertiamarin (II), and mangiferin (III) was observed at epoch 0, 75, and 40, respectively.

Moreover, RSM model was compared with the ANN model that represented the ANN model as more accurate method of interpolation, prediction, and validation. In contrast to the RSM, ANN model showed less deviation between the predicted and experimental values. Table 2 depicted the predicted values in response to the experimental values obtained for all the three studied bio-active compounds. In addition, comparison was drawn between two models on the basis of three significant statistical parameters, viz. Root mean square error (RMSE), Absolute average deviation (AAD) and regression coefficient (*r*^*2*^).

RMSE and AAD were calculated on the basis of Eqs. 4 and 5.

4 $$ \mathrm{RMSE}={\left(\frac{1}{\mathrm{n}}{\sum}_{\mathrm{i}=1}^{\mathrm{n}}{\left({Y}_{predicted}-{Y}_{experimental}\right)}^2\right)}^{1/2} $$

5 $$ \mathrm{AAD}\left(\%\right)=\left({\sum}_{\mathrm{i}=1}^{\mathrm{P}}\left(\left|{Y}_{i,\mathit{\exp}}-{Y}_{i, cal}\right|/{Y}_{i,\mathit{\exp}}\right)/P\right)\times 100 $$

Comparative overview of analytical parameters with RSM and ANN models are presented in Table 3. In present study, ANN demonstrated best validation statistical parameters, thus can be used as an accurate method in the optimization approaches.

**Table 3:** Comparison of response surface methodology (RSM) and artificial neural network (ANN) models

Parameters	Amarogentin (I)		Swertiamarin (II)		Mangiferin (III)	
	RSM	ANN	RSM	ANN	RSM	ANN
Root mean square error (RMSE)	0.004624	0.003351	0.046971	0.02104	0.03464	0.014931
Absolute average deviation (AAD)	0.089432	0.089124	1.296331	1.300059	0.572083	0.568817
Regression coefficient (r^2^)	99.8%	99.9%	99.9%	100%	99.7%	100%

#### Quantification of amarogentin, swertiamarin and mangiferin by HPTLC

**Fig. 6:**
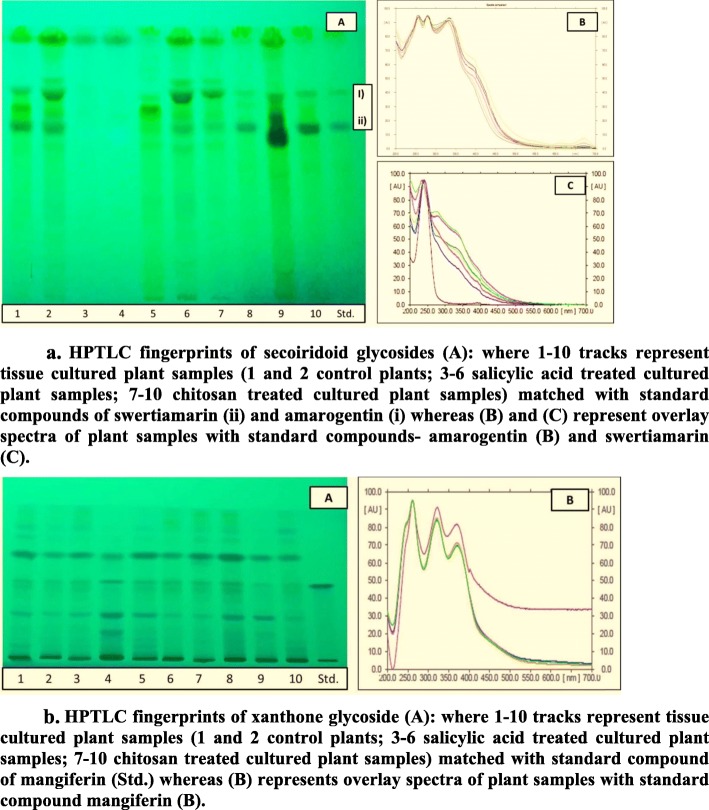
a. HPTLC fingerprints of secoiridoid glycosides (**a**): where 1–10 tracks represent tissue cultured plant samples (1 and 2 control plants; 3–6 salicylic acid treated cultured plant samples; 7–10 chitosan treated cultured plant samples) matched with standard compounds of swertiamarin (ii) and amarogentin (i) whereas (**b**) and (**c**) represent overlay spectra of plant samples with standard compounds- amarogentin (**b**) and swertiamarin (**c**). b. HPTLC fingerprints of xanthone glycoside (**a**): where 1–10 tracks represent tissue cultured plant samples (1 and 2 control plants; 3–6 salicylic acid treated cultured plant samples; 7–10 chitosan treated cultured plant samples) matched with standard compound of mangiferin (Std.) whereas (**b**) represents overlay spectra of plant samples with standard compound mangiferin (**b**)

High-performance thin-layer chromatography (HPTLC) is now emerging as an efficient, simple, specific, precise, and accurate as well as powerful analytical technique by which enormous samples can be analyzed at once. In the past few years, HPTLC technique has been proved to be a powerful technique for chemo-profiling in several medicinal plants [28, 29]. In present study, mobile phase (S1) gives clear dense spots on TLC plate with R_f_ of 0.62 for swertiamarin (II) and 0.8 for amarogentin (I) (Fig. 6a). Mobile phase (S2) gives clear single spot on TLC plate with R_f_ of 0.48 for mangiferin (III) (Fig. 6b). Peaks for all standards consistent to well-defined R_f_ were superimposable in 3-D densitogram patterns of all the test samples, and reference compounds (I), (II), and (III). Absorption spectra of (I), (II), and (III) compounds were compared with test samples to check the peak purity (Fig. 6). In HPTLC fingerprinting; track 6 and 9 in Fig. 6a (secoiridoids), whereas track 4 and 8 in Fig. 6b (mangiferin), display samples treated with optimal level of SA and CS concentrations (depicted higher concentrations of amarogentin, swertiamarin and mangiferin compounds). All the studied metabolites were quantified by means of the peak area parameter. HPTLC methods were validated for precision, accuracy and repeatability according to the ICH guidelines [30].

## Discussion

### Effect of PGR on organogenesis

In the present work, addition of auxin (NAA) with particular concentration of cytokinines (BAP or KN) in shoot inducing MS media resulted in healthy shoot formation.

In *Swertia*, BAP in combination with NAA and KN has been used most commonly for best organogenesis [31–33]. In many other medicinal plants, combination of cytokinines with auxins showed superior results for the shoot proliferation [8, 9]. The type and concentration of cytokinines and growth inducers affected the average number of shoots and mean length of shoots. In *Swertia* and other plants; most common plant growth regulator used in shoot elongation and multiplication is BAP in combination with various compositions of NAA and kinetin [8, 9, 31, 34].

In present research work, shoot cultures were used to check the effect of SA and CS on the production of secondary metabolites. In higher plants, secondary metabolites are often biosynthesized after the cell differentiation processes. As a result, organ cultures showed great prospective for the expression of active principles in plants. Likewise, in other genera of Gentianaceae family, Krstic et al. [35] described the higher production of xanthones and secoiridoids in organ cultures. Boroduske et al. [36] advocated shoot culture more suitable for secoiridoid production.

### Elicitor treatment

Exogenous treatment of lower concentration of SA elicits various significant secondary metabolites through upregulation of defense related gene expression [15, 18, 37]. In this study, lower concentration of SA (>5 mM) and CS (>10 mg L^− 1^) resulted in low content of compounds (Table 2, Fig. 6). Experimental design developed in the present study clearly showed that (9 mM) SA and (12 mg L^− 1^) CS yielded maximum content of amarogentin (0.435%), swertiamarin (4.987%), and mangiferin (4.357%) in shoot cultures of *S. paniculata*. However higher concentration of elicitors (A and B) treatment lowered the secoiridoid and xanthone contents (Table 2). In our previous report, likewise result was found in the wild samples of *S. paniculata* collected from higher altitudes of Himachal Pradesh and Uttarakhand [8].

It has been noted that unigenes are upregulated in aerial parts in comparison to the roots of *Swertia* for active metabolic synthesis of amarogentin, swertiamarin, and mangiferin [38]. Secoiridoids follow MVA/MEP pathway whereas xanthone glycosides follow phenylpropanoid signaling pathway [38–40]. Tissues having maximum secondary metabolite content have high level of gene expressions that can be triggered by optimal levels of precursors or elicitors [41]. Transcriptome studies revealed the elevated gene expressions involved in the metabolic pathways of secoiridoid and xanthone compounds. Research indicates that chitosan shows considerable increase of swertiamarin compound in *Centaurium erythraea* [36], amarogentin compound in *Swertia chirata* [12] and mangiferin compound in *Hypericum perforatum* cultured plants [42]. Chitosan is well known to release various defense genes and also to induce production of secondary metabolites in order to inhibit the growth of microbes [21, 43]. Krstić-Milošević et al. [44] studied the effect of salicylic acid and chitosan elicitors on the production of xanthones in hairy root clones of *Gentiana dinarica* Beck. and reported that highest concentrations of elicitors increases xanthone: aglycone norswertianin content, but simultaneously reduces the production of its glycoside: norswertianin-1-O-primeveroside. In another study, Tocci et al. [45] evaluated the effect of chitosan elicitation on xanthone biosynthesis in calli and in cell suspension cultures of *H. perforatum* subsp. *Angustifolium* and showed an increase in xanthone production (Paxanthone, 1,3,5,6-tetrahydroxyxanthone, 1,3,6,7-tetrahydroxyxanthone and cadensin G) in elicited cell cultures.

## Conclusion

This is the first study on the optimization of salicylic acid (SA) and chitosan (CS) for the production of amarogentin, swertiamarin and mangiferin in shoot cultures of *S. paniculata*. Central-composite design (CCD) of Response surface methodology (RSM) optimizes the linear, quadratic and interaction effects of SA and CS for maximum production of secoiridoid (amarogentin and swertiamarin) and xanthone (mangiferin) glycosides. RSM model was compared with the ANN model and ANN demonstrated more accurate method of interpolation and validation in contrast to RSM. To minimize the error and achieve faster convergence, ANN adopted back propagation algorithm during training phase for model training and convergence without any delay or loss. Shoot culture productivity can be enhanced by the optimal level of SA and CS treatment. These results endorse that advancement in tissue culture techniques could work for the production and enhancement of important secondary metabolites. Optimization of tissue culture system is the prerequisite for advancement of further biosynthetic potential of several cell / tissue culture types. In vitro propagation also provides the next platform for commercial plant cell lines and other biotechnological strategies. In future, chemical engineering and improvement in molecular techniques will provide the new dimensions to tissue culturing for increasing secondary metabolite production and use of bioreactors with optimized micropropagation techniques.

## Methods

### Chemicals and standard compounds

All chemicals and solvents used in present experimentation were of HPLC grade that have been purchased from E. Merck (Mumbai, India). All standards of amarogentin (1) (Catalogue No. CFN 90519; 98% purity) and swertiamarin (2) (Catalogue No. CFN 99818; 98% purity) were procured from Chromadex, India, and mangiferin (3) (Catalogue No. M3547-100MG;> 98% purity) was purchased from Sigma Aldrich. Modified Murashige and Skoog (MS) [46] medium supplemented with CaCl_2_ (332.2 mg/L)_,_ vitamins (Nitsch vitamin mixture), sucrose and agar, phytohormones, sterilizers, salicylic acid and chitosan (low molecular weight chitosan-derived from chitin shrimp with ≥75% deacetylation degree) were obtained from HiMedia™ (Mumbai, India).

### Plant material and micropropagation

Matured plants of *S. paniculata* were collected in fruiting stage from high altitude medicinal and aromatic plants nursery situated at Chakrata-Deoban region, Uttarakhand (30.798624° N, 77.780368° E; altitude 2600 m) during the month of November, 2017. The plant was authenticated on the basis of morphological characters by Prof. R. C Gupta, taxonomist expert. The Voucher specimens (No. 11112017) were prepared and deposited in the Department of Botany, Lovely Professional University, Phagwara, Punjab, India. The capsules were separated from the plants and seeds were separated, washed properly with distilled water to remove all the contaminants carried from the field. Seeds were stored in airtight container at 4 °C. One day before inoculation, seeds were soaked in 100 ppm GA_3_ (Gibberellic acid) overnight at 4 °C. For sterilization, seeds were dipped in 5% bavistin solution for 20 min; surface sterilized with 70% ethanol for 30 s and treated with 0.1% HgCl_2_ solution for 5 min with constant shaking, followed by 5 times washing with distilled water to remove the traces of the sterilizers. MS media were sterilized appropriately at the pressure of 15 psi and 121 °C temperature for 15 min.

For the germination of seedlings, seeds were inoculated with ½ MS medium having 3% (w/v) sucrose, vitamins, CaCl_2_ and 0.7% agar. After 6 weeks, small plantlets were transferred to the shoot inducing medium (SIM) with different combinations of BAP and KN (2.22 and 4.44 mM each) and NAA (2.60 and 5.20 mM) (Table 1). Mean shoot length developed with different combinations of auxins and cytokinines was estimated after 4 and 6 weeks. Healthy shoots (5–6 cm long) were developed within 6 weeks of culture period. After 6 weeks, multiplied shoots were taken out aseptically. Isolated shoots were treated with different concentrations of salicylic acid (1, 5, 10 and 20 mM) and chitosan (1, 5, 10 and 20 mg L^− 1^) mixed into ½ MS media for 2 weeks. All the experimental sets were conducted in flasks which were executed at the same time period to eliminate the culture conditions and environmental variations. Two control experimental sets (without elicitor treatment) were retained. All the experimentations were completed in triplicates. After 14 days, treated and untreated shoots were thoroughly washed with distilled water.

The pH of all the cultures was maintained at 5.8 using 1 M NaOH and 1 N HCl. All cultures and treatments were accomplished in 50–80% relative humidity, 25 ± 2 °C temperature and 16 h photoperiod (cool, white fluorescent light).

### Statistical optimization and experimental design by response surface methodology (RSM)

RSM was applied to check the effects of independent variables and their interaction on the % content of amarogentin, swertiamarin, and mangiferin. Two independent variables used in the experiment were salicylic acid (SA) (1–20 mM) and chitosan (CS) (1–20 mg L^− 1^). SA and CS concentrations were selected on the basis of preliminary studies. In RSM, central composite design (CCD) was applied at five different levels (−*a*, − 1, 0, + 1, +*a*) to obtain the individual and pairwise effects for elicitor treatment. *a* value is calculated by equation: 2^*(k-p)*/4^; k = no. of factors and p denotes replicate no. at central point. Total 13 experiments were conducted to test the five levels of SA and CS with full-factorial CCD. By using coded units, experimental and predicted values for the production of amarogentin, swertiamarin, and mangiferin in terms of the different variables of SA and CS are presented in Table 2.

Second order model (quadratic) equation was used to calculate the experimental response for secoiridoid and xanthone content as mentioned below:

6 $$ \mathrm{Y}={\upbeta}_0+{\upbeta}_1\mathrm{A}+{\upbeta}_2\mathrm{B}+{\upbeta}_{11}{\mathrm{A}}^2+{\upbeta}_{22}{\mathrm{B}}^2+{\upbeta}_{12}\mathrm{AB} $$

Where Y is the predicted response; A and B denote the level of variables; β_0_ is the scaling constant; β_1_ and β_2_ are linear coefficients; β_11_ and β_22_ are quadratic coefficients, and β_12_ depicts interaction coefficient. Response surface regression coefficient and Analysis of Variance (ANOVA) predicted the effects of independent variables on in vitro production % of amarogentin, swertiamarin, and mangiferin from *S. paniculata*.

### Artificial neural network (ANN) modeling

The functionality of ANN is to transform the given input vectors supplied to model into feature map or output with the help of specific rules. In the proposed approach, single layer, perceptron model was adopted which comprised of hidden neurons for generation of an approximate multilayer model. The predicted output computation is expressed with the help of Eq. (7) as:

7 $$ Y=f\left({A}_z\right)=\sum \limits_{p=1}^m{w}_{zp}\ast {x}_p+{\theta}_z $$

Here, *Y* represents the ouput obtained from output layer, *f*(*A*_*z*_) denoted the activation functions which is responsible for non-linear nature of model associated with neuron z. *w*_*zp*_ represents weight connection between neuron z and p. *θ*_*z*_ denotes the input bias and *x*_*p*_ illustrated the inputs given to neuron *p*.

To minimize the error and to achieve faster convergence, ANN adopted back propagation algorithm during training phase for model training and convergence without any delay or loss. Due to consideration of appropriate neuron size, the obtained results are sound and accurate without any compromises.

**Fig. 7:**
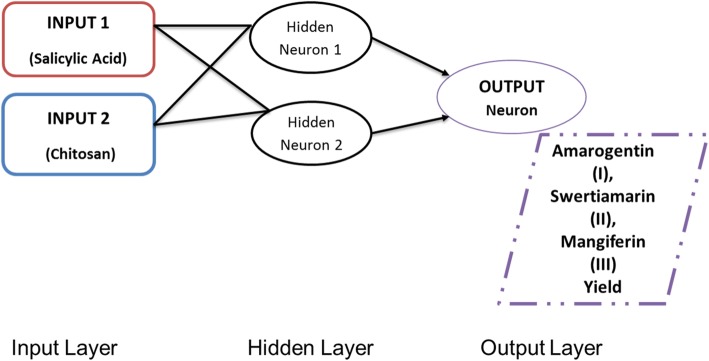
ANN Architecture

The multilayer perceptron model consists of two inputs (salicyclic acid and chitosan), one hidden and output layer for prediction (Fig. 7). The output layer will be different for three distinguished outputs as amarogentin, swertiamarin, and mangiferin as shown in Table 2. For training and validation analysis, Log Sigmoidal function is used as an activation unit for non- linear output prediction. For synaptic weight adjustment and analysis training data is trained with help of Marquardt algorithm and validation is supported and performed with the help of 5 fold cross validation strategy.

### Sample preparation

In vitro grown material of *S. paniculata* was thoroughly washed with tap water to remove the growth hormone traces and was then dried completely in the shade at room temperature. Well dried in vitro samples were finely powdered in a mixer grinder separately (Champ Essentials, Morphy Richards, India). One gram powdered samples was extracted separately using microwave assisted extraction (MAE) with 50% aqueous ethanol solvent (2× 20 mL) [4]. All the extracts were filtered separately through Whatman no: 1 filter paper, centrifuged at 6000 rpm (at 4 °C for 5 min.) and then evaporated to dryness with a Rota-evaporator. Dried extracts were dissolved in methanol solvent to make the final volume (mg mL^− 1^) and then tubes containing extracts were stored in refrigerator at 4 °C for further phytochemical analysis.

Preparation of Standard Solution: Stock solutions of amarogentin, swertiamarin compounds (10 mg each) were dissolved in 10 mL methanol (mg ml^− 1^) and 5 mg mangiferin standard was dissolved in 50 ml methanol (0.1 mg ml^− 1^).

### Quantitative determination of marker compounds by HPTLC

The HPTLC system was CAMAG (Muttenz, Switzerland) having Linomat-5 automatic sample applicator and CAMAG TLC scanner-3 provided with CATS software (version: 1.4.4.6337) furnished with a 100 μL Hamilton syringe (with fixed 100 nl/s delivery rate). Chromatography was performed on stationary phase composed of 20 cm × 10 cm pre-coated silica gel 60 F_254_ HPTLC plates (with 0.25 mm thickness). Samples were administered to the plates as 5 mm wide bands with Hamilton syringe. 3 μL plant samples were loaded on chromatographic plate.

Separation was carried out on thin-layer chromatography aluminium plate pre-coated with silica gel 60 F_254,_ eluted with ethyl acetate: methanol: water (77:15:8 v/v/v) mobile phase (S1) for the quantification of amarogentin [I] and swertiamarin [II] compounds [47] whereas with ethyl acetate: glacial acetic acid: formic acid: water (100:11.0:11.0:26 v/v) mobile phase (S2) for the quantification of mangiferin [III] compound [48]. After development up to 75 mm in twin-trough glass tank (CAMAG), plates were dried with hot air dryer and clear bands (without any post-chromatographic derivatization) were visualized under UV (UV cabinet with dual wavelength UV lamp) at λ = 254 nm. Immediately plates were scanned at 254 nm reflectance wavelength with CAMAG TLC Scanner. Densitometric scanning conditions were set at 4.00 × 0.30 mm slit dimension with 20 mm/s scanning speed and 100 μm/step data resolution. The R_f_ value of (I), (II) and (III) in mobile systems with reference standards and *Swertia* crude samples were resolved and separated at 254 nm. All the HPTLC experimentation was conducted at 25 (±2 °C) temperatures with 40% relative humidity.

Linearity range of the stock solution of each marker compound (amarogentin, swertiamarin and mangiferin) was calibrated with the application of 2, 4, 6, 8, 10, 12 μL amount range applied on HPTLC plate. Calibration curve was generated with peak area versus relevant concentration. Regression equation and corresponding peak area was used to calculate the yield of all the three reference compounds in all tested plant samples.

### Statistical analysis

MINITAB 18.0 software program (Minitab Inc., State College, PA, USA); MATLAB software and Microsoft Excel 2010 (Ver. 14.0.7228.5000) were used in statistical optimization and analysis.

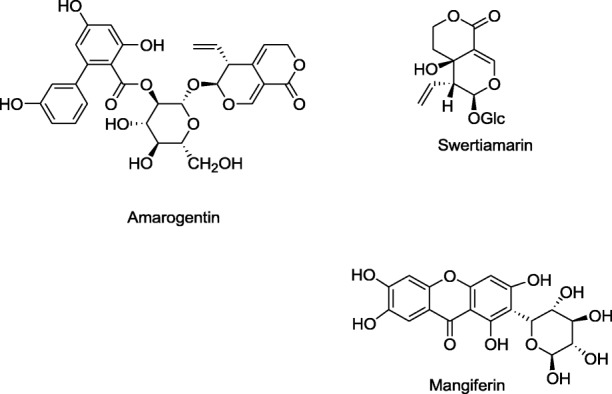



## Supplementary information

**Additional file 1: Table S1.** Response Surface Regression: % amarogentin (I), swertiamarin (II) and mangiferin (III) versus salicylic acid (SA), chitosan (CS). **Table S2.** Analysis of Variance for % of amarogentin (I), swertiamarin (II) and mangiferin (III) compounds.

## Data Availability

The datasets used and/or analysed during the current study available from the corresponding author on reasonable request.

## Abbreviations

HPTLC: High performance thin layer chromatography;
SA: Salicylic acid
CS: Chitosan
ANN: Artificial neural network
RSM: Response surface methodology
CCD: Central composite design
BAP: 6-Benzylaminopurine
KN: Kinetin
NAA: 1-Naphthaleneacetic acid

## References

[1] Li J, Zhao YL, Huang HY, Wang YZ (2017). Phytochemistry and pharmacological activities of the genus *Swertia* (Gentianaceae): a review. Am J Chin Med.

[2] Kumar V, Van Staden J (2016). A review of *Swertia chirayita* (Gentianaceae) as a traditional medicinal plant. Front Pharmacol.

[3] Negi JS, Singh P, Rawat B (2011). Chemical constituents and biological importance of *Swertia*: a review. Curr Res Chem.

[4] Negi J. S., Bisht V. K., Singh P., Rawat M. S. M., Joshi G. P. (2013). Naturally Occurring Xanthones: Chemistry and Biology. Journal of Applied Chemistry.

[5] Negi JS, Singh P, Pant GJ, Rawat MS (2010). RP-HPLC analysis and antidiabetic activity of *Swertia paniculata*. Nat. Prod. Commun..

[6] Pant N, Misra H, Jain DC (2014). A xanthone glycoside from aerial parts of *Swertia paniculata*. J Saudi Chem Soc.

[7] Kaur P, Gupta RC, Dey A, Pandey DK (2019). Simultaneous quantification of oleanolic acid, ursolic acid, betulinic acid and lupeol in different populations of five *Swertia* species by using HPTLC-densitometry: comparison of different extraction methods and solvent selection. Ind Crop Prod.

[8] Kaur P, Pandey DK, Gupta RC, Dey A (2019). Assessment of genetic diversity among different population of five *Swertia* species by using molecular and phytochemical markers. Ind Crop Prod.

[9] Mittal J, Sharma MM (2017). Enhanced production of berberine in In vitro regenerated cell of *Tinospora cordifolia* and its analysis through LCMS QToF. 3. Biotech..

[10] Sharma V, Kamal B, Srivastava N, Negi Y, Dobriyal AK, Jadon VS (2015). Enhancement of in vitro growth of *Swertia chirayita* Roxb. Ex Fleming co-cultured with plant growth promoting rhizobacteria. Plant Cell Tissue Organ Cult.

[11] Kawakami H, Hara K, Komine M, Yamamoto Y (2015). Production of secoiridoids by adventitious root culture of *Swertia japonica*. In Vitro Cell Dev Biol Plant.

[12] Keil M, Härtle B, Guillaume A, Psiorz M (2000). Production of amarogentin in root cultures of *Swertia chirata*. Planta Med.

[13] Trivellini A, Lucchesini M, Maggini R, Mosadegh H, Villamarin TS, Vernieri P, Mensuali-Sodi A, Pardossi A (2016). Lamiaceae phenols as multifaceted compounds:bioactivity, industrial prospects and role of “positive-stress”. Ind Crop Prod.

[14] Mendoza D, Cuaspud O, Arias JP, Ruiz O, Arias M (2018). Effect of salicylic acid and methyl jasmonate in the production of phenolic compounds in plant cell suspension cultures of *Thevetia peruviana*. Biotechnol Rep.

[15] Ghasemzadeh A, Jaafar H, Karimi E (2012). Involvement of salicylic acid on antioxidant and anticancer properties, anthocyanin production and chalcone synthase activity in ginger (*Zingiber officinale* roscoe) varieties. Int J Mol Sci.

[16] Hari G, Vadlapudi K, Vijendra PD, Rajashekar J, Sannabommaji T, Basappa G (2018). A combination of elicitor and precursor enhances psoralen production in *Psoralea corylifolia* Linn. Suspension cultures. Ind Crop Prod.

[17] Jiao W, Li X, Wang X, Cao J, Jiang W (2018). Chlorogenic acid induces resistance against *Penicillium expansum* in peach fruit by activating the salicylic acid signaling pathway. Food Chem.

[18] Obinata N, Yamakawa T, Takamiya M, Tanaka N, Ishimaru K, Kodama T (2003). Effects of salicylic acid on the production of procyanidin and anthocyanin in cultured grape cells. Plant Biotechnol.

[19] Jaisi A, Panichayupakaranant P (2017). Chitosan elicitation and sequential Diaion® HP-20 addition a powerful approach for enhanced plumbagin production in *Plumbago indica* root cultures. Process Biochem.

[20] Kamalipourazad M, Sharifi M, Maivan HZ, Behmanesh M, Chashmi NA (2016). Induction of aromatic amino acids and phenylpropanoid compounds in *Scrophularia striata* Boiss. Cell culture in response to chitosan-induced oxidative stress. Plant Physiol Biochem.

[21] Lucini L, Baccolo G, Rouphael Y, Colla G, Bavaresco L, Trevisan M (2018). Chitosan treatment elicited defence mechanisms, pentacyclic triterpenoids and stilbene accumulation in grape (*Vitis vinifera* L.) bunches. Phytochemistry.

[22] Malayaman V, Sisubalan N, Senthilkumar RP, Ranjithkumar R (2017). Chitosan mediated enhancement of hydrolysable tannin in *Phyllanthus debilis* Klein ex Willd via plant cell suspension culture. Int J Biol Macromol.

[23] John RP, Sukumaran RK, Nampoothiri KM, Pandey A (2007). Statistical optimization of simultaneous saccharification and L (+)-lactic acid fermentation from cassava bagasse using mixed culture of lactobacilli by response surface methodology. Biochem Eng.

[24] Leonard J, Seth B, Sahu BB, Singh VR, Patra N (2018). Statistical optimization for enhanced bacoside a production in plant cell cultures of *Bacopa monnieri*. Plant Cell Tissue Organ Cult.

[25] Bezerra MA, Santelli RE, Oliveira EP, Villar LS, Escaleira LA (2008). Response surface methodology (RSM) as a tool for optimization in analytical chemistry. Talanta..

[26] Amdoun R, Benyoussef EH, Benamghar A, Khelifi L (2019). Prediction of hyoscyamine content in *Datura stramonium* L. hairy roots using different modeling approaches: response surface methodology (RSM), artificial neural network (ANN) and Kriging. Biochem Eng..

[27] Dey A, Bhattacharya R, Mukherjee A, Pandey DK (2017). Natural products against Alzheimer's disease: Pharmaco-therapeutics and biotechnological interventions. Biotechnol Adv.

[28] Arumugam T, Kumar PS, Gopinath KP (2017). HPTLC fingerprint profile, in vitro antioxidant and evaluation of antimicrobial compound produced from Brevibacillus brevis-EGS9 against multidrug resistant *Staphylococcus aureus*. Microb Pathog.

[29] Saraswathi VS, Rajaguru P, Santhakumar K (2017). Solar catalysed activity against methyl orange dye, cytotoxicity activity of MCF-7 cell lines and identification of marker compound by HPTLC of Lagerstroemia speciosa. J Photochem Photobiol.

[30] ICH Harmonised Tripartite Guideline. Validation of Analytical Procedures: Text and Methodology Q2(R1). Geneva: International Conference on Harmonisation of Technical Requirements for Registration of Pharmaceuticals for Human Use; 2005. p.1–13. https://www.gmpcompliance.org/guidemgr/files/Q2(R1).pdf.

[31] Balaraju K, Saravanan S, Agastian P, Ignacimuthu S (2011). A rapid system for micropropagation of *Swertia chirata* Buch-ham. Ex wall.: an endangered medicinal herb via direct somatic embryogenesis. Acta Physiol Plant.

[32] Pant M, Bisht P, Gusain MP (2012). In vitro propagation through root-derived callus culture of *Swertia chirata* Buch.-ham. Ex wall. Afr J Biotechnol.

[33] Wang L, An L, Hu Y, Wei L, Li Y (2009). Influence of phytohormones and medium on the shoot regeneration from leaf of *Swertia chirata* Buch.-ham. Ex wall. In vitro. Afr J Biotechnol.

[34] Chaudhuri RK, Pal A, Jha TB (2007). Production of genetically uniform plants from nodal explants of *Swertia chirata* Buch. Ham. Ex wall—an endangered medicinal herb. In Vitro Cell Dev Biol Plant..

[35] Krstić D, Janković T, Šavikin-Fodulović K, Menković N, Grubiŝić D (2003). Secoiridoids and xanthones in the shoots and roots of *Centaurium pulchellum* cultured in vitro. In Vitro Cell Dev Biol.

[36] Boroduske A, Nakurte I, Tomsone S, Lazdane M, Boroduskis M, Rostoks N (2016). In vitro culture type and elicitation affects secoiridoid and xanthone LC–ESI–TOF MS profile and production in *Centaurium erythraea*. Plant Cell Tissue Organ Cult.

[37] Baenas N, Ferreres F, García-Viguera C, Moreno DA (2015). Radish sprouts-characterization and elicitation of novel varieties rich in anthocyanins. Food Res Int.

[38] Rai A, Nakamura M, Takahashi H, Suzuki H, Saito K, Yamazaki M (2016). High-throughput sequencing and de novo transcriptome assembly of *Swertia japonica* to identify genes involved in the biosynthesis of therapeutic metabolites. Plant Cell Rep.

[39] Liu Y, Wang Y, Guo F, Zhan L, Mohr T, Cheng P, Huo N, Gu R, Pei D, Sun J, Tang L (2017). Deep sequencing and transcriptome analyses to identify genes involved in secoiridoid biosynthesis in the Tibetan medicinal plant *Swertia mussotii*. Sci Rep.

[40] Padhan JK, Kumar V, Sood H, Singh TR, Chauhan RS (2015). Contents of therapeutic metabolites in *Swertia chirayita* correlate with the expression profiles of multiple genes in corresponding biosynthesis pathways. Phytochemistry.

[41] Pal T, Padhan JK, Kumar P, Sood H, Chauhan RS (2018). Comparative transcriptomics uncovers differences in photoautotrophic versus photoheterotrophic modes of nutrition in relation to secondary metabolites biosynthesis in *Swertia chirayita*. Mol Biol Rep.

[42] Valletta A, De Angelis G, Badiali C, Brasili E, Miccheli A, Di Cocco ME, Pasqua G (2016). Acetic acid acts as an elicitor exerting a chitosan-like effect on xanthone biosynthesis in *Hypericum perforatum* L. root cultures. Plant Cell Rep.

[43] Pichyangkura R, Chadchawan S (2015). Biostimulant activity of chitosan in horticulture. Sci Hortic.

[44] Krstić-Milošević D, Janković T, Uzelac B, Vinterhalter D, Vinterhalter B (2017). Effect of elicitors on xanthone accumulation and biomass production in hairy root cultures of *Gentiana dinarica*. Plant Cell Tiss Org.

[45] Tocci N, Ferrari F, Santamaria AR, Valletta A, Rovardi I, Pasqua G (2010). Chitosan enhances xanthone production in *Hypericum perforatum* subsp. *angustifolium* cell cultures. Nat Prod Res.

[46] Murashige T, Skoog F (1962). A revised medium for rapid growth and bio assays with tobacco tissue cultures. Physiol Plant.

[47] Bhandari P, Gupta A, Singh B, Kaul V (2006). HPTLC determination of swertiamarin and amarogentin in *Swertia* species from the Western Himalayas. JPC-J Planar Chromat..

[48] Pandey DK, Basu S, Jha TB (2012). Screening of different east Himalayan species and populations of *Swertia* L. based on exomorphology and mangiferin content. Asian Pac J Tropical Biomed.

