# Supplementary material for: Optimization of salicylic acid and chitosan treatment for bitter secoiridoid and xanthone glycosides production in shoot cultures of Swertia paniculata using response surface methodology and artificial neural network
Source: BMC Plant Biol. 2020 May 19;20:225. doi: 10.1186/s12870-020-02410-7 (PMC7238632; doi:10.1186/s12870-020-02410-7)
Supplement: Supplementary file 1 — Additional file 1: Table S1. Response Surface Regression: % amarogentin (I), swertiamarin (II) and mangiferin (III) versus salicylic acid (SA), chitosan (CS). Table S2. Analysis of Variance for % of amarogentin (I), swertiamarin (II) and mangiferin (III) compounds. [file 12870_2020_2410_MOESM1_ESM.docx]

Table S1. Response Surface Regression: % amarogentin (I), swertiamarin (II) and mangiferin (III) versus salicylic acid (SA), chitosan (CS)

| **Term** | **Amarogentin (I)** | | | **Swertiamarin (II)** | | | **Mangiferin (III)** | | |
| --- | --- | --- | --- | --- | --- | --- | --- | --- | --- |
|  | **Coeff. ±**  **S.E** | **t** | **P˃(t)** | **Coeff. ±**  **S.E** | **t** | **P˃(t)** | **Coeff. ±**  **S.E** | **t** | **P˃(t)** |
| Constant | 0.42800 ± 0.0028 | 152.100 | 0.000^**^ | 4.87040 ± 0.0286 | 169.884 | 0.000^**^ | 4.28860 ± 0.0211 | 202.814 | 0.000^**^ |
| A | 0.05000 ± 0.0022 | 22.475 | 0.000^**^ | 1.01422 ± 0.0226 | 44.749 | 0.000^**^ | 0.22064 ± 0.0167 | 13.198 | 0.000^**^ |
| B | 0.04096  ± 0.0022 | 18.414 | 0.000^**^ | 0.68208 ± 0.0226 | 30.094 | 0.000^**^ | 0.51145 ± 0.0167 | 30.595 | 0.000^**^ |
| A*A | -0.09500 ± 0.0023 | -39.822 | 0.000^**^ | -1.07083 ± 0.0243 | -44.057 | 0.000^**^ | -0.45055 ± 0.0179 | -25.133 | 0.000^**^ |
| B*B | -0.08100 ± 0.0023 | -33.953 | 0.000^**^ | -1.13207 ± 0.0243 | -46.577 | 0.000^**^ | -0.53055 ± 0.0179 | -29.595 | 0.000^**^ |
| A*B | 0.01200 ± 0.0031 | 3.814 | 0.007^*^ | 0.09375 ± 0.0320 | 2.925 | 0.022^*^ | 0.06500 ± 0.0236 | 2.749 | 0.029^*^ |

where Coeff= Regression Coefficient; S.E = Standard Error of Regression Coefficient;

^**^ Very significant, where p˂ 0.001 and ^*^Significant, where p˂ 0.05

Table S2. Analysis of Variance for % of amarogentin (I), swertiamarin (II) and mangiferin (III) compounds.

| **Source** | **df** | **Amarogentin (I)** | | | **Swertiamarin (II)** | | | **Mangiferin (III)** | | |
| --- | --- | --- | --- | --- | --- | --- | --- | --- | --- | --- |
|  |  | **SS** | **F** | **P** | **SS** | **F** | **P** | **SS** | **F** | **P** |
| Regression | 5 | 0.130094 | 657.19 | 0.000^**^ | 26.9328 | 1310.74 | 0.000^**^ | 5.48632 | 490.80 | 0.000^**^ |
| Linear | 2 | 0.033423 | 422.10 | 0.000^**^ | 11.9511 | 1454.05 | 0.000^**^ | 2.48211 | 555.12 | 0.000^**^ |
| Square | 2 | 0.096095 | 1213.5 | 0.000^**^ | 14.9466 | 1818.51 | 0.000^**^ | 2.98731 | 668.11 | 0.000^**^ |
| Interaction | 1 | 0.000576 | 14.55 | 0.007^*^ | 0.0352 | 8.55 | 0.022^*^ | 0.01690 | 7.56 | 0.029^*^ |
| Residual Error | 7 | 0.000277 |  |  | 0.0288 |  |  | 0.01565 |  |  |
| Lack-of-Fit | 3 | 0.000147 | 1.51 | 0.341 | 0.0085 | 0.55 | 0.672 | 0.00594 | 0.82 | 0.548 |
| Pure Error | 4 | 0.000130 |  |  | 0.0203 |  |  | 0.00971 |  |  |
| Total | 12 | 0.130371 |  |  | 26.9616 |  |  | 5.50197 |  |  |
| R^2^ |  | 99.64% |  |  | 99.82% |  |  | 99.51% |  |  |

where df= degree of freedom; SS= Sum of squares;

^**^ Very significant, where p˂ 0.001 and ^*^Significant, where p˂ 0.05
